# B Cells Are Required to Generate Optimal Anti-Melanoma Immunity in Response to Checkpoint Blockade

**DOI:** 10.3389/fimmu.2022.794684

**Published:** 2022-05-26

**Authors:** Shubhra Singh, Jason Roszik, Neeraj Saini, Vipul Kumar Singh, Karishma Bavisi, Zhiqiang Wang, Long T. Vien, Zixi Yang, Suprateek Kundu, Richard E. Davis, Laura Bover, Adi Diab, Sattva S. Neelapu, Willem W. Overwijk, Kunal Rai, Manisha Singh

**Affiliations:** ^1^Department of Genomic Medicine, The University of Texas MD Anderson Cancer Center, Houston, TX, United States; ^2^Department of Melanoma Medical Oncology, The University of Texas MD Anderson Cancer Center, Houston, TX, United States; ^3^Department of Lymphoma/Myeloma, The University of Texas MD Anderson Cancer Center, Houston, TX, United States; ^4^Department of Pathology and Genomic Medicine, Houston Methodist Research Institute, Houston, TX, United States; ^5^Department of Immunology, The University of Texas MD Anderson Cancer Center, Houston, TX, United States; ^6^Department of Biostatistics, The University of Texas, Health Science Center, Houston, TX, United States; ^7^Department of Biostatistics, The University of Texas MD Anderson Cancer Center, Houston, TX, United States; ^8^Nektar Therapeutics, San Francisco, CA, United States

**Keywords:** knockout (KO), programmed death-ligand 1 (PD-L1), cytotoxic T-lymphocyte-associated protein 4 (CTLA-4), the Cancer Genome Atlas melanoma (TCGA), immunotherapy, Blymphocytes, chemokineCXCL13, toll-like receptor 7/8

## Abstract

Immunotherapies such as checkpoint blockade therapies are known to enhance anti-melanoma CD8^+^ T cell immunity, but only a fraction of patients treated with these therapies achieve durable immune response and disease control. It may be that CD8^+^ T cells need help from other immune cells to generate effective and long-lasting anti-tumor immunity or that CD8^+^ T cells alone are insufficient for complete tumor regression and cure. Melanoma contains significant numbers of B cells; however, the role of B cells in anti-melanoma immunity is controversial. In this study, B16 melanoma mouse models were used to determine the role of B cells in anti-melanoma immunity. C57BL/6 mice, B cell knockout (KO) C57BL/6 mice, anti-CD19, and anti-CXCL13 antibody-treated C57BL/6 mice were used to determine treatment efficacy and generation of tumor-specific CD8^+^ T cells in response to PD-L1 blockade alone or combination with TLR-7/8 activation. Whole transcriptome analysis was performed on the tumors from B cell depleted and WT mice, untreated or treated with anti-PD-L1. Both CD40-positive and CD40-negative B cells were isolated from tumors of TLR-7/8 agonist-treated wild-type mice and adoptively transferred into tumor-bearing B cell KO mice, which were treated with anti-PD-L1 and TLR-7/8 agonist. Therapeutic efficacy was determined in the presence of activated or inactivated B cells. Microarray analysis was performed on TLR-7/8-treated tumors to look for the B cell signatures. We found B cells were required to enhance the therapeutic efficacy of monotherapy with anti-PD-L1 antibody and combination therapy with anti-PD-L1 antibody plus TLR-7/8 agonist. However, B cells were not essential for anti-CTLA-4 antibody activity. Interestingly, CD40-positive but not CD40-negative B cells contributed to anti-melanoma immunity. In addition, melanoma patients’ TCGA data showed that the presence of B cell chemokine CXCL13 and B cells together with CD8^+^ T cells in tumors were strongly associated with improved overall survival. Our transcriptome data suggest that the absence of B cells enhances immune checkpoints expression in the tumors microenvironment. These results revealed the importance of B cells in the generation of effective anti-melanoma immunity in response to PD-1-PD-L1 blockade immunotherapy. Our findings may facilitate the design of more effective anti-melanoma immunotherapy.

## Introduction

Melanoma is the most deadly form of skin cancer (1) and is estimated to have caused 60,712 deaths worldwide in 2018 (2) and 100,350 new cases, and 11,480 deaths in the United States in 2020 (3). Although immunotherapies have emerged as the most promising treatments for metastatic melanoma (4) most patients with melanoma do not respond to available immunotherapies.

The finding that the number of tumor-infiltrating CD8^+^ T cells was strongly associated with patient survival led to T cell-based immunotherapy for melanoma. Cancer vaccines have been shown to increase the activity of T cells that recognize tumor-associated antigens, but robust clinical responses remain anecdotal (5). Similarly, promising checkpoint blockade therapies (anti-CTLA-4 and anti-PD-1) are known to enhance T cell immunity, but only a fraction of patients treated with these therapies achieve durable immune response and disease control (6). It may be that CD8^+^ T cells need help from other immune cells to generate effective and long-lasting anti-tumor immunity or that CD8^+^ T cells alone are insufficient for complete tumor regression and cure. The importance of interactions between B cells and T cells in autoimmunity (7, 8) and transplant (9) suggests that B cells may have a role in generating anti-tumor CD8^+^ T cell immunity.

In this study, we found that B16 melanoma responds better to monotherapy with anti-PD-L1 antibody and to combination therapy with anti-PD-L1 antibody plus TLR-7/8 agonist in the presence of B cells. Furthermore, CD40-positive B cells were required to generate optimal anti-melanoma immunity in response to the combination therapy.

## Methods

### Mice and Cell Lines

All animal experiments were performed under National Institutes of Health guidelines and approved by the Institutional Animal Care and Use Committee of The University of Texas MD Anderson Cancer Center. C57BL/6 mice were purchased from The Jackson Laboratory. Female mice were used at 6–8 weeks of age. Mycoplasma-free B16.F10 and B16.OVA melanoma cells were obtained from the American Type Culture Collection and maintained in RPMI 1640 supplemented with 10% heat-inactivated FBS, L-glutamine, sodium pyruvate, nonessential amino acids, and penicillin-streptomycin (all from Invitrogen/Life Technologies).

### TLR-7/8 Agonist and Its Vehicle

An injectable formulation of the TLR-7/8 agonist (3M-052) and the vehicle was provided by 3M Drug Delivery Systems (3M Center, St. Paul, MN) (10).

### Tumor Induction, Treatment, and Monitoring

C57BL/6 mice or C57BL/6 mice B cell knockout (KO) (muMt^-^) or anti-CXCL13 antibody (MAB470, R&D system), anti-CD19 antibody (BE0150, bio-x-cell) treated C57BL/6 mice were s.c. inoculated with 400,000 B16.F10 or B16.OVA melanoma cells for tumor development. On days 4, 7, 10, and 14 after tumor cell inoculation, mice were treated with i.p. injection of 200 μg of anti-CTLA-4 (9H10) or 200 μg of anti-PD-L1 (10F.9G2) or their isotype control antibodies (all from Bio X Cell). On days 8, 12, and 16 after tumor cell inoculation, mice were treated with intratumoral injection of 35 μg of TLR-7/8 agonist.

Three days before tumor inoculation, mice were treated with i.p. injection of 200 μg of anti-CXCL13/anti-CD19 antibody or their isotype control Abs. Treatment was repeated every 3 days.

In an adoptive transfer experiment, wild-type (WT) C57BL/6 mice with B16.F10 were treated with TLR-7/8 for 24 hr. Then tumors were isolated, and tumor-infiltrating CD40^+^ and CD40^–^ B cells were sorted by flow cytometry and then adoptively transferred to tumor-bearing B cell KO mice. Twenty-four hours after cell implant, the mice were treated with anti-PD-L1 and TLR-7/8 agonist according to the schedule described in the preceding paragraph.

Tumor size was expressed as the product of perpendicular diameters of tumors measured with calipers. Mice were sacrificed when tumor size reached 200 mm^2^. Results of survival experiments were analyzed using a log-rank Mantel-Cox test.

### Flow Cytometry Analysis

Leukocytes were isolated from mechanically disrupted tumors by using lymphocyte separation medium (Corning Cellgro). Antigen-specific T cell responses were evaluated by using OVA tetramer (clone H-2Kb SIINFEKL, Beckman Coulter) and CD8 (53-6.7, 1:200 dilution, BioLegend) staining. Intracellular Treg staining was performed using the Cytofix/Cytoperm kit (BD Biosciences). Cells were stained with antibodies against CD45 (30-F11, 1:200), CD19 (ID3,1:200), and CD40 (3-23,1:200) (all from Bio Legend); CD4 (GK1.5, 1:200, from BD Pharmingen); CD25 (PC61, 1:200, BD Bioscience); and FoxP3 (FJK-16s,1:200, Invitrogen). Dead cells were excluded from analysis using a fixable live stain (LIVE/DEAD fixable aqua stain, 1:150 dilution, Thermo Fisher Scientific). Data were acquired on an LSR II flow cytometer (BD Biosciences) and analyzed using FlowJo software, version 10.

### Cytokine/Chemokine Assay

Tumors were mechanically disrupted and centrifuged, and the supernatants were collected. An aliquot of 25 μl of a tumor supernatant from each sample was used to perform the assay. Cytokines/chemokines were measured using a Milliplex mouse cytokine/chemokine panel (Millipore) according to the manufacturer’s instructions. A fluorescence signal was measured on a Luminex 100/200 system, and data were analyzed using GraphPad Prism 8 software.

### ELISA-Based Cells Assay

B16 tumor-bearing B cell KO and WT mice were treated intratumorally with 3M-052 or vehicle on days 8 and 12 after tumor cell inoculation. On days 5 and 11 after treatment, serum was collected and analyzed for the presence of B16 tumor-specific serum IgG antibodies by ELISA-based cells assay. Briefly, 150,000 B16 cells per well suspended in 40 ul of PBS were coated and dried ON onto a 96 plate. On the day of the assay, wells were blocked with PBS-2% FBS for 1 hr. After blocking, serum dilutions were added as a source of primary antibodies and incubated for 1 hr. The plate was washed 5 times with PBS-T (PBS containing Tween). 50 ul of secondary antibody, goat IgG anti-mouse, HRP conjugated (1:2000) was added to each well and incubated for 1 hour. After washing 5 times, the substrate solution (TMB) was added to each well and incubated for 30 minutes. The reaction was stopped with H2SO4 0.2N, and the OD absorbance values were read at 450 nm.

### Microarray

Total RNA of TLR-7/8 agonist-treated or vehicle-treated tumors draining lymph nodes were extracted using the RNAqueous kit (Ambion). The RNA quality was checked by using a Bioanalyzer 2100 instrument (Agilent). One hundred nanograms of total RNA was amplified and biotin-labeled through a two-round Eberwine procedure using MessageAmp II and Illumina TotalPrep RNA amplification kits (Ambion) and hybridized to Illumina Ref-6 version 2 mouse whole-genome arrays. Hierarchical clustering and heat mapping were performed using Cluster and Treeview software from Eisen et al. (11).

### Whole Transcriptome Analysis

B16 melanoma bearing B cells depleted, and WT mice were treated or untreated with anti-PD-L1 antibody (every three days). After 3 days of third treatment, total RNA from tumors was extracted using Qiagen RNeasy Mini Kit (Cat# 74104). The quality of the RNA was assessed with using the Agilent RNA 6000 nano kit (Cat# 5067-1511). Approximately 1 ug of total RNA was used in conjunction with the Vazyme Ribo-off rRNA removal kit (Human/Mouse/Rat, Cat# N406-01) to specifically deplete all rRNA species. cDNA libraries were constructed using Ion Total RNA-Seq Kit v2 from ThermoFisher (Cat# 4479789) and manufacturers recommended protocol. Briefly, 10-50 ng of rRNA removed RNA was fragmented with RNAase III and purified using Nucleic Acid binding beads from Thermofisher (Cat# 4479681). RNA sequencing and sequence alignment were performed by PrimBio Research Institute LLC (Exton, PA). Statistical analysis was performed using the Moderated T-test to determine significant differentially expressed genes based on the replicates of each condition.

### Statistical Analysis

All results are expressed as mean ± SEM. For therapeutic experiments, 8 to 10 mice were assigned per treatment group. Data were analyzed using unpaired Student’s t-test, and ANOVA by using GraphPad Prism software (version 9), and differences were considered to be significant at P<0.05. The tumor volume was analyzed at different time points by using unpaired Student’s t-test. The analysis data set was the mice from the last available data point for which 90% of mice were still alive in the particular group comparison. Results of survival experiments were analyzed using a log-rank Mantel-Cox test. All experiments were performed at least twice with comparable results.

## Results

### B Cells Enhance Anti-Melanoma Immunity in Response to Monotherapy With Anti-PD-L1 Antibody

**Figure 1:**
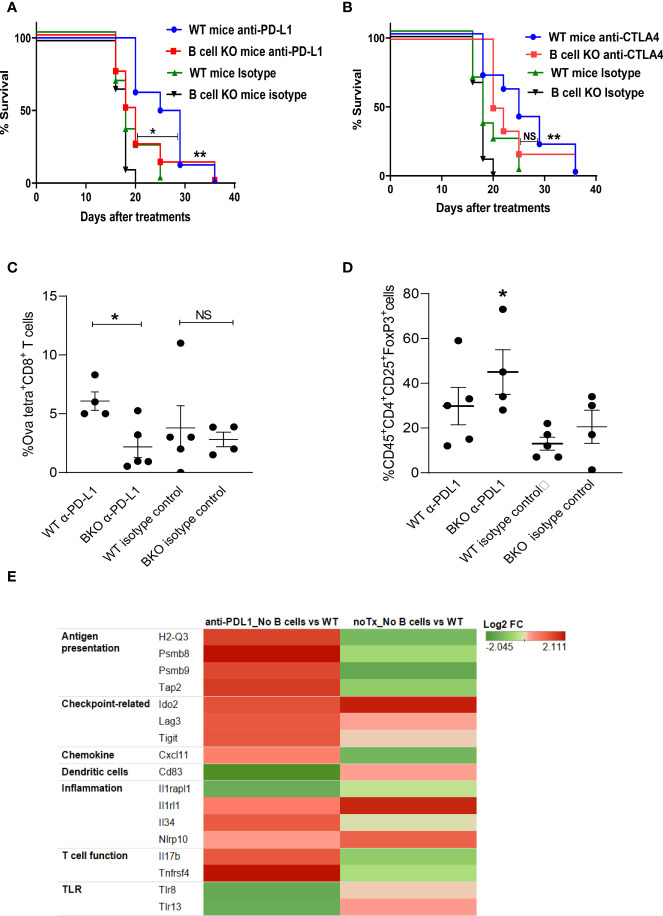
B cells play a role in anti-PD-L1 but not anti-CTLA-4–mediated anti-melanoma immunity. **(A, B)** WT and B cell KO (BKO) mice bearing B16.F10 tumors (400,000 cells/tumor) were treated with α-PD-L1 or α-CTLA-4 on days 4, 7, 10, and 14 after tumor cell inoculation. Kaplan-Meier survival curves, each with 8 to 10 mice per group. *P < 0.05, **P < 0.01, log-rank test. NS, not significant. **(C)** Mice bearing B16. Ova tumors were treated as indicated. Graph shows % of OVA-tetramer^+^CD8^+^ T cells in tumor **(D)** Tregs in WT and B cell KO (BKO) mice, after three days of the 3rd anti-PDL1 or isotype antibody treatment, n = 4 to 5 mice per group. *P < 0.05, unpaired Student’s t-test for tumor-specific CD8^+^ T cells and one-way ANOVA for Tregs. **(E)** Heatmap (transcriptome analysis) of B16 melanoma from B cell depleted and WT mice, treated or untreated with anti-PD-L1. The graph depicts fold changes in genes expression in anti-PD-L1 treated B cell depleted vs WT mice and untreated B cell depleted vs WT mice (n=4 mice/group).

To determine whether B cells play any role in generating anti-melanoma immunity in response to checkpoint blockade, tumor-bearing WT and B cell KO mice were treated with anti-PD-L1 or anti-CTLA-4 antibodies. We found that both anti-PD-L1 and anti-CTLA-4 antibodies prolonged mouse survival compared to isotype control antibodies (**Figures 1A, B**). Interestingly, after anti-PD-L1 treatment, survival was longer in WT mice than in B cell KO mice, indicating that B cells enhance anti-PD-L1 antibody activity, but after anti-CTLA-4 treatment, there was no difference in survival between WT mice and B cell KO mice (**Figures 1A, B**) In addition, we found more tumor-specific CD8^+^ T cells in anti-PD-L1-treated WT mice than in anti-PD-L1-treated B cell KO mice (**Figure 1C**). In contrast, we found more regulatory T cells in anti-PD-L1-treated B cell KO mice than in any other groups of mice (**Figure 1D**). The gating strategies of **Figures 1C, D** are shown in **Supplementary Figure 1**.

To further confirm our findings and identify differences in tumor immune cell infiltration between B cell-depleted and WT mice, we performed a transcriptome analysis of tumors after anti-PD-L1 treatment. Interestingly, we found upregulation of antigen-presenting immune cells and activated T cell-related markers in B cell-depleted mice compared to WT mice (**Figure 1E**). However, in accordance with our flow cytometry data (**Figure 1D**), transcriptome data showed higher expression of regulatory T cell (Treg) markers such as indolamine-2,3-dioxygenase (IDO2), lymphocyte-activation gene 3 (LAG-3), and ITIM domains (TIGIT) (**Figure 1E**) in B cell-depleted mice than in WT mice. Inflammation-related genes such as IL-33 receptors (IL1rl1) and Nlrp10 were also upregulated in B cell-depleted mice than in WT mice (**Figure 1E**).

### B Cells Enhance Anti-Melanoma Immunity in Response to Combination Therapy With Anti-PD-L1 Antibody and TLR-7/8 Agonist

**Figure 2:**
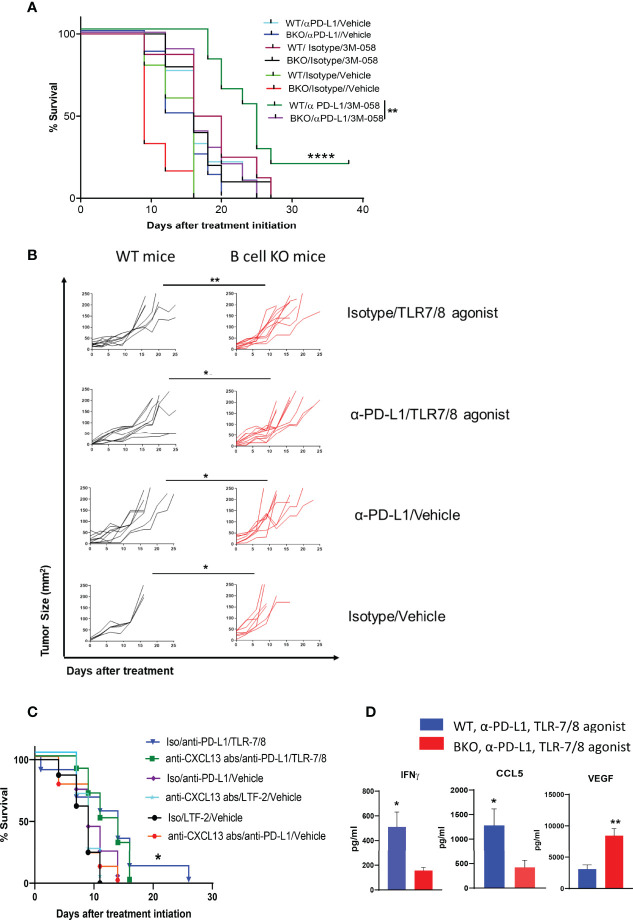
B cells enhance anti-melanoma immunity mediated by combination therapy with α-PD-L1 plus TLR-7/8 agonist. WT, B cell KO (BKO), and anti-CXCL13 antibody treated mice bearing B16.F10 tumors (400,000 cells/tumor) were treated with α-PD-L1 or isotype control on days 7, 10, 14 and 18 after tumor cell inoculation and/or intratumoral injection of TLR-7/8 or vehicle on days 8, 12, and 16 after tumor cell inoculation. **(A, C)** Kaplan-Meier survival curves. ****P < 0.0001, **P < 0.01, *P < 0.05, log-rank test. LTF-2, isotype control antibody; NS, not significant. **(B)** Tumor size. *P < 0.05, **P < 0.01 unpaired Student’s t-test (5-10 mice per group). **(D)** Cytokine levels. *P < 0.05, **P < 0.01, unpaired Student’s t-test (5 mice per group).

Previously, we reported that PD-1 pathway blockade enhanced the therapeutic efficacy of injectable TLR-7/8 agonist (12) against melanoma. We sought to determine whether B cells play an essential role in the anti-melanoma immune response mediated by combination therapy with anti-PD-L1 antibody and TLR-7/8 agonist. Indeed, we found that B cell KO mice generated significantly milder responses to anti-PD-L1 antibody, TLR-7/8 agonist, or the combination of TLR-7/8 agonist plus anti-PD-L1 antibody than WT mice, as demonstrated by shorter survival (**Figure 2A**) and larger tumor size (**Figure 2B**) of the B cell KO mice. Tumor size at particular time points is shown in **Supplementary Figure 2A**. Microarray data of draining lymph nodes of TLR-7/8 agonist–treated and vehicle-treated mice tumors showed significantly higher upregulation of CXCL13 in TLR-7/8–treated mice than in vehicle-treated mice (**Supplementary Figure 2B**). Indeed, we found B cell chemokine CXCL13 was required to generate optimal anti-melanoma effect and prolonged mouse survival in response to combination therapy with anti-PD-L1 plus TLR-7/8 (**Figure 2C**).

Furthermore, among the mice treated with the combination therapy, levels of Th1 cytokine IFNγ and T-cell-recruiting chemokine CCL5 in the tumor were significantly lower in the B cell KO mice than in the WT mice, whereas the level of VEGF was significantly higher in the B cell KO mice (**Figure 2D**).

### CD40-Positive B Cells Prolong Mouse Survival in Response to Combination Therapy

**Figure 3:**
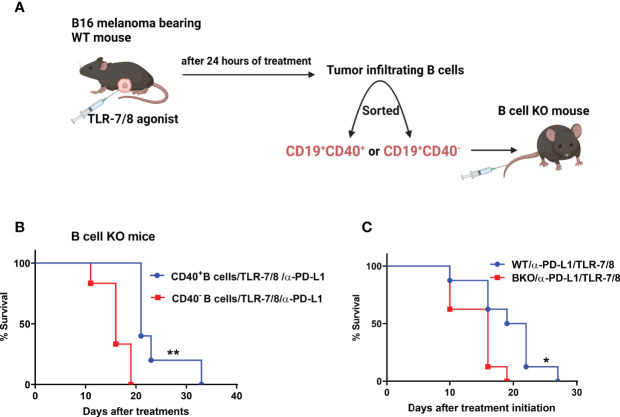
CD40-positive B cells enhance the therapeutic efficacy of anti-PD-L1 plus TLR-7/8 agonist therapy. **(A)** Experimental design. **(B)** B cell KO mice were treated with TLR-7/8 agonist plus anti-PD-L1 and received either 5000 CD19^+^CD40^-^ B cells or 5000 CD19^+^CD40^+^ B cells. **(C)** WT and B cell KO mice were treated with TLR-7/8 agonist plus anti-PD-L1. Kaplan-Meier survival curves, each with 5 to 6 mice per group. *P < 0.05, **P < 0.01, log-rank test.

Next, we determined whether only CD40-positive B cells play an essential role in anti-melanoma immunity. We previously showed that TLR-7/8 agonist induced B cell activation and upregulation of CD40 on the B cell surface (12) Both CD19^+^CD40^+^ and CD19^+^CD40^-^ B cells were isolated from tumors of TLR-7/8 agonist–treated WT mice (**Figure 3A**). Then CD40-positive or CD40-negative B cells were injected into the tail vein of B cell KO mice, and mice were treated with the combination of TLR-7/8 agonist plus anti-PD-L1. As expected, CD40-positive B cell receivers had longer survival than CD40-negative B cell receivers in response to combination therapy (**Figure 3B**). Indeed, CD40-positive B cell receivers’ survival was similar to that of WT mice, and CD40-negative B cell receivers’ survival was similar to that of B cell KO mice (**Figures 3B, C**). Furthermore, in another set of experiments, we found a higher percentage of B cells and activated B cells in tumor-draining lymph nodes of anti-PD1 antibody-treated mice than PBS treated mice (**Supplementary Figure 3**).

### B Cell Chemokine CXCL13 and B Cells Together With CD8 T Cells in Tumors Associate With Melanoma Patients’ Survival

**Figure 4:**
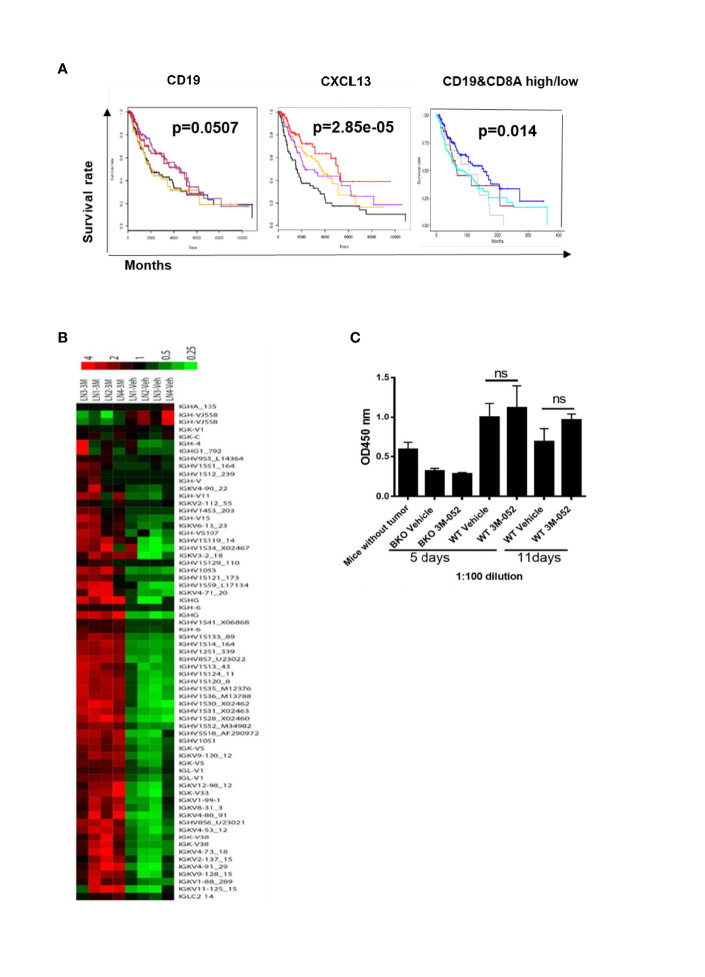
**(A)** Presence of B cells (CD19+), B cell chemokine CXCL13, or B cells together with CD8+ T cells in tumors is positively associated with melanoma patients’ survival. Kaplan-Meier plots show survival by quartiles of CXCL13 and CD19 mRNA expression (black, lowest 25%; orange, next 25%; purple, next 25%; red, highest 25%) and combinations of CD19 and CD8A mRNA expression (blue, CD19 high CD8A high; grey, CD19 high CD8A low; brown, CD19 low CD8A high; cyan, CD19 low CD8A low). High expression was defined as above, and low expression was defined as below the median gene expression. The P-values were derived from log-rank tests. **(B)** Microarray of tumor-draining lymph nodes after 4 days of treatment with TLR-7/8 agonist (3M or 3M-052) or vehicle (Veh). Shown are genes of the variable or constant region of Ig heavy or light chain (4 mice/group). **(C)** B16-specific IgG antibodies in serum in response to treatment with TLR-7/8 agonist (3M-052) or vehicle; unpaired Student’s t-test (5 mice/group). NS, not significant.

B cells were previously found to be activated in human cancer patients (13). We used TCGA analysis to determine the association between B cells and clinical outcomes of patients with melanoma. Gene expression and clinical data from the melanoma TCGA project were downloaded from public TCGA repositories. Kaplan-Meier analysis was used to generate plots of overall survival according to mRNA expression by RNA sequencing. Kaplan-Meier plots were generated using the “survival” R package (14). We found that the presence of B cells (CD19^+^) or B cell chemokine CXCL13 in tumors was associated with improved overall survival among melanoma patients. Interestingly, the presence of B cells together with CD8^+^ T cells in tumors was also associated with improved survival (**Figure 4A**). These findings indicated that tumor-infiltrating B cells contribute to the generation of an anti-melanoma immune response.

### B Cells Play an Antibody-Independent Role in TLR-7/8 Agonist-Mediated Anti-Melanoma Immunity

We performed a microarray analysis of draining lymph nodes of TLR-7/8 agonist–treated tumors to look for the B cell gene signature. We found multifold upregulation of genes of the variable or constant region of antibody heavy or light chain in TLR-7/8 agonist–treated mice compared to vehicle-treated mice (**Figure 4B**).

Next, we determined whether 3M-052-activated B cells produce tumor-specific antibodies responsible for tumor cell killing. B16 tumor-bearing B cell KO and WT mice were treated intratumorally with TLR-7/8 agonist or vehicle; serum was collected at various time points and analyzed for the presence of B16 tumor-specific serum IgG antibodies by ELISA as described under methods. Interestingly, a higher level of B16-specific IgG antibodies was not observed in the serum of TLR-7/8 agonist–treated mice compared with the serum of vehicle-treated mice (**Figure 4C**). These results indicated a potential antibody-independent role of B cells in TLR-7/8 agonist-mediated anti-melanoma immunity.

## Discussion

Although melanoma’s immunogenic nature makes this disease susceptible to immunotherapy, metastatic melanoma remains highly resistant to established immunotherapies like cancer vaccines and checkpoint blockade therapies. This highlights the need to understand the molecular mechanism of immunotherapy-induced anti-melanoma immune responses to develop new approaches for effective treatment. B cells enhance CD8^+^ T cell-mediated immunity during autoimmunity and allograft rejection (9, 15). Still, the role of B cells in anti-tumor CD8^+^ T cell immunity and in cancer patient survival is not well studied. Studies suggested that tumor-infiltrating B cells play a dual role, exhibiting both pro-tumor and anti-tumor activity.

Our study demonstrated that B cells enhance the anti-melanoma immunity in response to checkpoint blockade therapy. To the best of our knowledge, this is the first preclinical study that shows the involvement of B cells in anti-tumor activity in response to anti-PD-L1 antibody therapy. Furthermore, our findings showed that combination therapy with anti-PD-L1 antibody and TLR-7/8 agonist also required CD40 positive B cells and their chemokine CXCL13 to generate optimum anti-melanoma immunity. These results suggest that the interaction between CD40 on B cells and CD40L on other immune cells activates the CD40 pathway, which stimulates effective anti-tumor T cell immunity. Indeed, we found that the absence of B cells significantly reduced tumor-specific immunity.

Interestingly, we found that several antibodies -related genes were highly upregulated in response to TLR-7/8 agonist treatment, but we did not find the antibody-dependent killing of melanoma cells. These results suggest that B cells act as antigen-presenting cells, it may be that CD40 expression makes them potent antigen-presenting cells in the tumor microenvironment.

Our results are in accordance with DiLillo et al.’s findings that B cell-depleted mice showed a higher tumor burden of subcutaneous melanoma and lung metastasis than WT mice (16). Similarly, Kobayashi et al. found that B cells enhance anti-melanoma immunity in the B16.F10 melanoma model (17). Recently, some clinical studies also showed a key role of B cells in response to immune checkpoint blockade therapy (18, 19). Helmink et al. showed that the presence of B cells within tertiary lymphoid structures was associated with response to immune checkpoint blockade therapy (18). In contrast to these findings, other studies demonstrated an immune-suppressive function of B cells (20, 21), or no role of B cells in immunotherapy response (22). The controversial role of B cells in anti-melanoma immunity may be due to different phenotypes of B cells and different frequencies of B cells in the tumor microenvironment. Our study suggests that activated B cells or CD40-positive B cells are crucial to enhance anti-melanoma immunity.

Our transcriptome data suggest that the absence of B cells enhances Tregs/immune checkpoints and inflammation in tumors and these changes suppress anti-PDL1-induced anti-tumor immune response and reduce mouse survival.

Although our results reveal the role of B cells in enhancing the anti-melanoma immunity in response to immunotherapy, these results must be interpreted with caution, and a few limitations should be borne in mind, such as; using only one tumor model in the study and found limited contribution of B cells in anti-melanoma immunity.

We expect our findings to lead the field closer to the development of biomarkers based on B cell activation that predicts response to immunotherapy. In addition, our results may facilitate the design of more effective anti-melanoma immunotherapy.

## Data Availability Statement

The original contributions presented in the study are publicly available. This data can be found here: https://www.ncbi.nlm.nih.gov/geo/, GSE190261 and GSE202879.

## Ethics Statement

The animal study was reviewed and approved by All animal experiments were performed under National Institutes of Health guidelines and approved by the Institutional Animal Care and Use Committee of The University of Texas MD Anderson Cancer Center.

## Author Contributions

Conception, design and study supervision: MS, Development of methodology: MS, SS, VS, KB, JR, ZW, LV, RD, LB, ZY, SK Resources and critical comments on manuscript: KR, SSN, WO, AD, NS, Writing the manuscript: MS and SS.

## Funding

This work was supported by Department of Defense Idea Award CA160521 (to M.S.), The University of Texas MD Anderson Cancer Center Specialized Programs of Research Excellence in Melanoma (P50CA093459 to MS) and National Institutes of Health/National Cancer Institute Grant P30CA016672, which supports the flow cytometry facility at MD Anderson Cancer Center.

## Conflict of Interest

Author WO was employed by company Nektar Therapeutics.

The remaining authors declare that the research was conducted in the absence of any commercial or financial relationships that could be construed as a potential conflict of interest.

## Publisher’s Note

All claims expressed in this article are solely those of the authors and do not necessarily represent those of their affiliated organizations, or those of the publisher, the editors and the reviewers. Any product that may be evaluated in this article, or claim that may be made by its manufacturer, is not guaranteed or endorsed by the publisher.

## Abbreviations

KO, knockout; PD-L1, Programmed death-ligand 1; PD-1, Programmed cell death protein 1; CTLA-4, cytotoxic T-lymphocyte-associated protein 4; TCGA, The Cancer Genome Atlas.
