# Supplementary material for: B Cells Are Required to Generate Optimal Anti-Melanoma Immunity in Response to Checkpoint Blockade
Source: Front Immunol. 2022 May 26;13:794684. doi: 10.3389/fimmu.2022.794684 (PMC9204262; doi:10.3389/fimmu.2022.794684)
Supplement: Supplementary file 1 [file DataSheet_1.pdf]

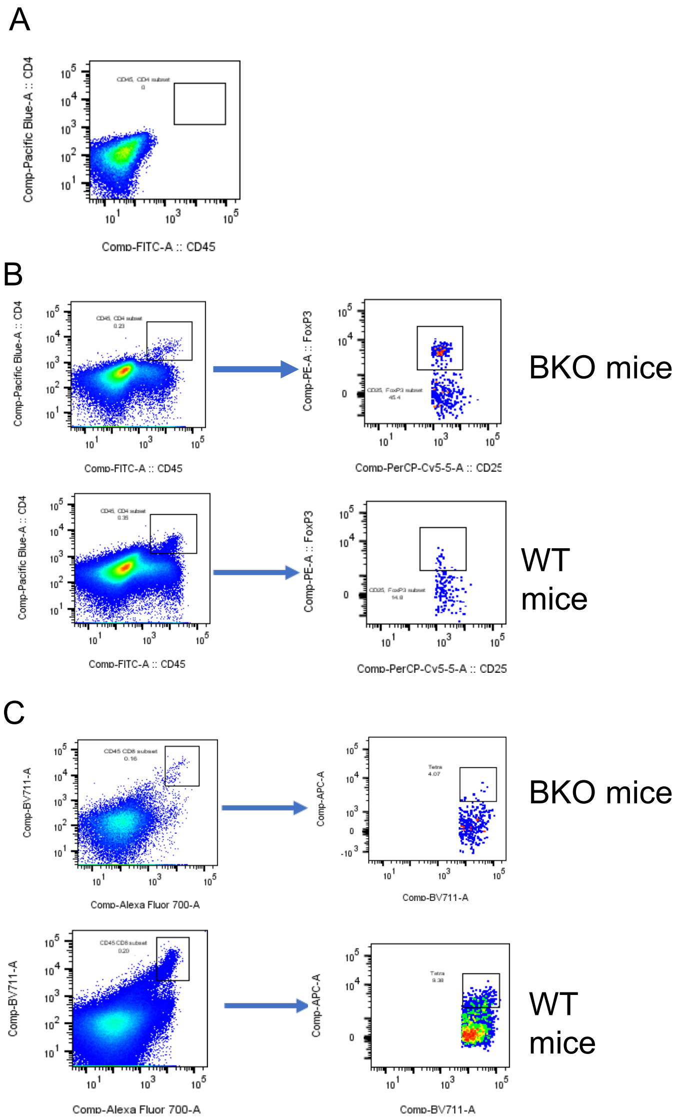

Supplemental Figure 1. Gating strategy of flowcytometry analysis, Tumor bearing, B- cell KO (BKO) and WT mice were treated with anti-PDL1 and tumor infiltrated leukocytes were analyzed for the presence of Tregs and tumor specific $CD8^+$ T cells (Ova tetramer). Graphs represent (A) unstained control (B) $CD25^+FoxP3^+$ gated on $CD45^+CD4^+$ (C) $CD8^+$Ova tetramer$^+$ gated on $CD45^+CD8^+$

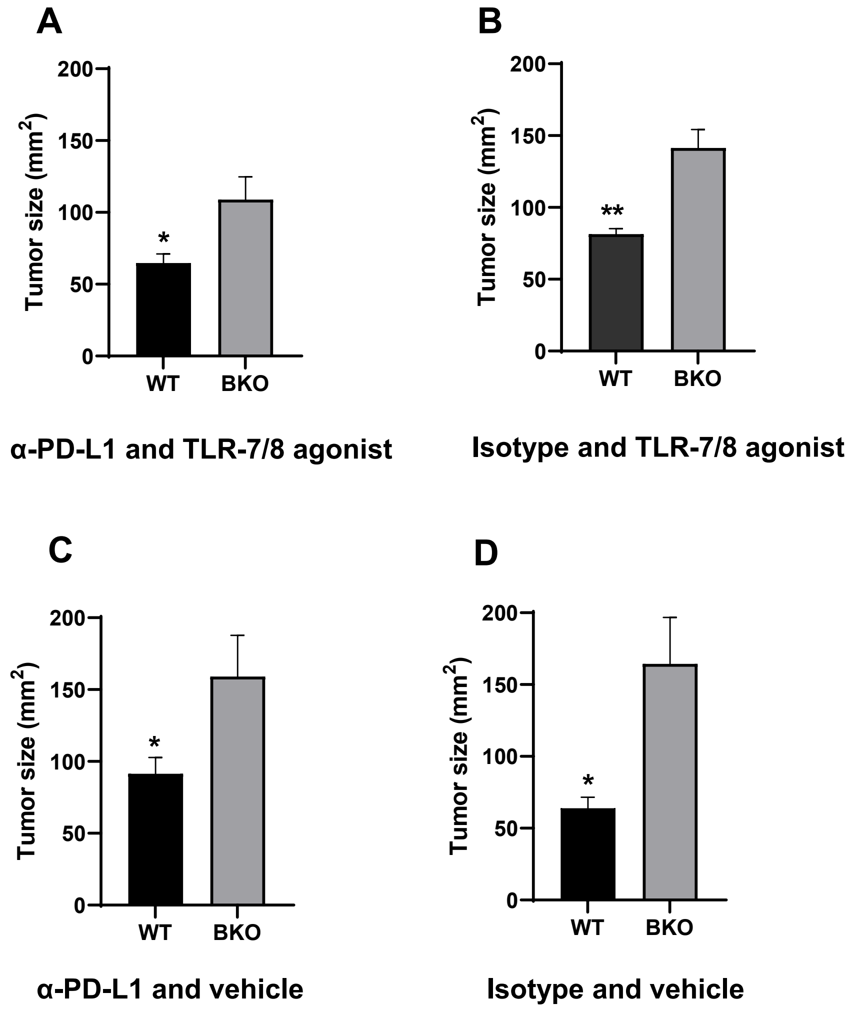

Supplemental Figure. 2A. Tumor size on day 12 (A,B,C) and on day 9 (D). Mice were treated as indicated *P<0.05, **P<0.01, unpaired Student's t-test . BKO, B cell knockout

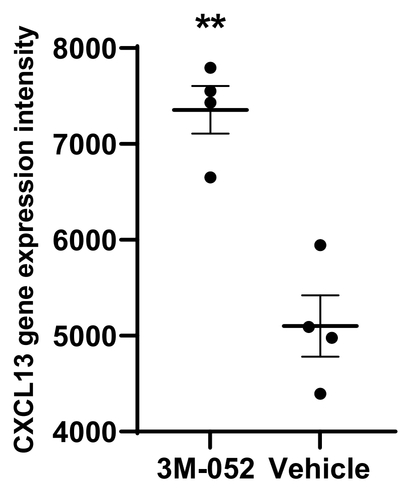

Supplemental Figure. 2B. Upregulation of CXCL13 in response to TLR-7/8 agonist (3M-052). Microarray of tumor-draining lymph nodes after 4 days of treatment with 3M-052 or vehicle. **P<0.01, unpaired Student's t-test .

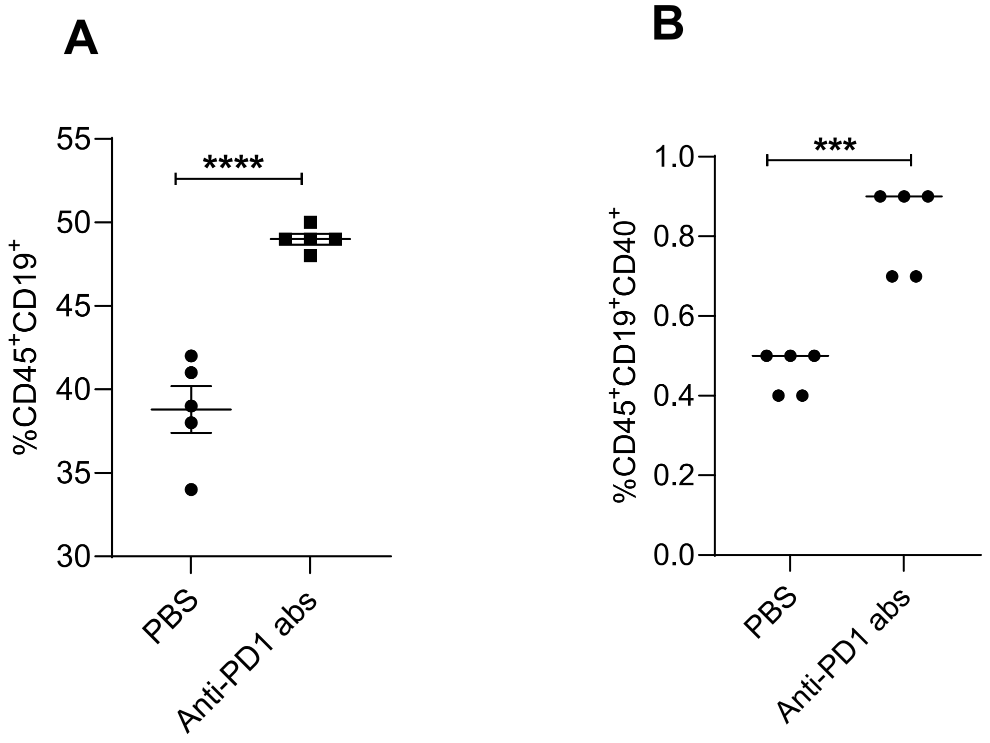

Supplemental Figure 3. B cells (A) and activated B cells (B) in the tumor-draining lymph node. Tumor-bearing, WT mice were treated with anti-PD1 antibody (100ug/mouse) or PBS intraperitoneally every other day, and lymph nodes cells were analyzed for the presence of CD45+CD19+ and CD45+CD19+CD40+ by flow cytometry.***P=0.0002 and .****P=0.0001 , unpaired Student's t-test
